# Die Rolle der Epidemiologie in der SARS-CoV-2-Pandemie: Bedeutung, Aufgaben, Methoden und Erkenntnisse

**DOI:** 10.1007/s00103-021-03399-6

**Published:** 2021-09-03

**Authors:** Iris Pigeot, Sabine Gleich, Linus Grabenhenrich

**Affiliations:** 1 Leibniz-Institut für Präventionsforschung und Epidemiologie – BIPS, Abteilung Biometrie und EDV, Universität Bremen, Achterstr. 30, 28359 Bremen, Deutschland; 2 Fachbereich Mathematik und Informatik, Universität Bremen, Bibliothekstr. 5, 28359 Bremen, Deutschland; 3 Landeshauptstadt München, Gesundheitsreferat, Gesundheitsschutz, Hygiene und Umweltmedizin, Bayerstr. 28 a, 80335 München, Deutschland; 4 Robert Koch-Institut, Nordufer 20, 13353 Berlin, Deutschland

Die SARS-CoV-2-Pandemie stellt nun bereits seit März 2020 die Gesundheitssysteme, die Gesellschaft und die Wirtschaft weltweit vor extreme Herausforderungen. Selbst nachdem verschiedene Impfstoffe zugelassen wurden und zumindest in Deutschland aktuell (Juli 2021) schon etwa die Hälfte der Bevölkerung vollständig geimpft werden konnte, entschärft sich die Situation bislang nicht wesentlich, da immer wieder neue und zum Teil deutlich infektiösere Varianten das Pandemiegeschehen beherrschen. Trotz der Entwicklung effektiver Impfstoffe wird uns die Pandemie vermutlich noch für längere Zeit begleiten.

Damit stellt sich auch die Frage nach geeigneten Maßnahmen zur Bekämpfung der Pandemie immer wieder neu. Um diese jedoch einleiten zu können, sind Informationen zu den Ausbreitungs- und Übertragungswegen, zum Infektionsgeschehen und zu Prävalenz, Inzidenz, Letalität und Risikofaktoren von COVID-19 und deren Folgen erforderlich. Die mithilfe dieser Informationen zu entwickelnden Modelle des Infektionsgeschehens erlauben, die Auswirkungen verschiedener Maßnahmen zu verstehen und zu prognostizieren.

In Deutschland wurden zahlreiche Studien durchgeführt und epidemiologische Daten erhoben. Dennoch scheint eine bessere Koordination und eine stärker auf frei zugängliche Daten ausgerichtete Forschungsdateninfrastruktur erforderlich zu sein, um den Beitrag der Epidemiologie zur Kontrolle dieser und zukünftiger Pandemien zu optimieren. Ein wichtiger Schritt in diese Richtung ist die Initiative zum Aufbau einer Nationalen Forschungsdateninfrastruktur (NFDI), in deren Rahmen u. a. das Konsortium NFDI4Health (Nationale Forschungsdateninfrastruktur für personenbezogene Gesundheitsdaten) gefördert wird. Als Teil dieses Konsortiums widmet sich die NFDI4Health Task Force COVID-19 (s. auch Beitrag von *Schmidt et al.* in diesem Heft) dem Ziel, den Zugang zu COVID-19-bezogenen Forschungsergebnissen im Gesundheitsbereich zu erleichtern. In diesem Kontext ist die Idee zu diesem Themenheft^1^ entstanden.

Insgesamt beleuchten 13, in 3 übergeordnete Themenbereiche gruppierte Beiträge verschiedene Aspekte der Epidemiologie dieser pandemischen Viruserkrankung. In den ersten 5 Beiträgen werden u. a. epidemiologische Grundlagen zu den Ausbreitungswegen, Methoden der Modellierung und verschiedene Studientypen aufbereitet. In den anschließenden 3 Beiträgen werden die wichtigsten allgemeinen Erkenntnisse zum Infektionsgeschehen in Deutschland sowie zu den Risikofaktoren und zu den Risikogruppen für schwere Krankheitsverläufe dargestellt. Die letzten 5 Beiträge befassen sich mit spezifischen Fragestellungen anhand ausgewählter Bevölkerungsgruppen.

Im ersten Beitrag von *Oh et al.* werden mögliche Übertragungswege des SARS-CoV‑2 aus Sicht des Fremd- und Eigenschutzes diskutiert. Es wird speziell darauf eingegangen, wie lange jemand infektiös ist, wie das Virus ausgeschieden und aufgenommen wird und wie es sich in der Gesellschaft verbreitet. Dabei wird deutlich, dass die asymptomatische Aerosolübertragung und Überdispersion von SARS-CoV‑2 eine nicht zu unterschätzende Rolle bei der Weitergabe des Erregers spielen. Daher ist es erforderlich, geeignete epidemiologische Modelle an der Hand zu haben, mit denen die Auswirkung von Maßnahmen zur Eindämmung der Pandemie untersucht werden kann.

In dem Artikel von *Priesemann et al.* wird entsprechend der Beitrag epidemiologischer Modelle zur Beurteilung zentraler Aspekte des Pandemieverlaufs, wie z. B. Reproduktionszahl, Dunkelziffer und Infektionssterblichkeit, sowie zur Quantifizierung der Wirkung von Maßnahmen und der Effekte der Test-Trace-Isolate-Strategie dargestellt. Der bei der Beurteilung des Ausbreitungsgeschehens wichtige Aspekt der Dunkelziffer wird in dem Beitrag von *Fiedler et al.* erneut aufgegriffen. Dort wird ein Modell vorgestellt, das bereits in der frühen Phase einer Pandemie in der Lage ist, nicht gemeldete Fallzahlen effizient zu schätzen. Die Autor:innen berechnen für Mitte April 2020 in Deutschland insgesamt 2,8-mal so viele Infektionen wie die Zahl der registrierten Fälle und setzen diese ins Verhältnis zu den entsprechenden Zahlen in Italien.

In dem anschließenden Beitrag von *Zeeb et al.* wird die Bedeutung epidemiologischer Studien für das Verständnis der SARS-CoV-2-Pandemie herausgearbeitet. Dabei werden Beispielfragestellungen anhand von in Deutschland und international durchgeführten Studien vorgestellt und die jeweiligen epidemiologischen Ansätze diskutiert, aber auch Versäumnisse bei der Erhebung erforderlicher Daten beschrieben sowie die Notwendigkeit einer stärker auf frei zugängliche Daten ausgerichteten Forschungsinfrastruktur betont.

Dem Aspekt einer verbesserten Forschungsdateninfrastruktur widmen sich *Schmidt et al.* explizit in ihrem Artikel zur NFDI4Health Task Force COVID-19, die COVID-19-bezogene klinische, epidemiologische und Public-Health-Forschung unter Berücksichtigung der FAIR-Prinzipien („Findability, Accessibility, Interoperability, Re-Usability“) leichter zugänglich machen und eine schnellere Kommunikation von Ergebnissen befördern will.

In dem ersten der folgenden 3 Beiträge teilen *Schilling et al.* den Pandemieverlauf in Deutschland 2020 in 4 epidemiologisch verschiedene Phasen ein. In Phase 0 wurden überwiegend sporadische Fälle unter 60 Jahren und regional begrenzte Ausbrüche beobachtet, in Phase 1 (1. Welle) vermehrt Ausbrüche in Krankenhäusern, Pflegeeinrichtungen sowie ein größerer Anteil an älteren und schwer erkrankten Fällen, in Phase 2 durch Reisen bedingte COVID-19-Fälle im Alter von 15–59 Jahren und vereinzelt größere Ausbrüche in Betrieben und in Phase 3 (2. Welle) deutlich mehr schwere Fälle in allen Altersgruppen.

Insgesamt haben sich bis zum 19.04.2021 über 3,1 Mio. Menschen in Deutschland infiziert, die sich aber nicht über alle Bevölkerungsgruppen gleichmäßig verteilen, wie *Koppe et al.* in ihrem Beitrag aufzeigen. Besonders betroffen waren z. B. bestimmte Berufsgruppen oder Personen, die an Großveranstaltungen teilgenommen haben. Zudem beschreiben die Autor:innen Komorbiditäten, die verstärkt zu schweren Krankheitsverläufen geführt haben.

Komplementär zeigt der Beitrag von *Dragano et al.* auf, dass auch sozioökonomische Faktoren die Ausbreitung von SARS-CoV‑2 deutlich beeinflusst haben: Ab November 2020 wiesen Kreise mit einer geringen Wohnfläche je Einwohner ausgeprägt höhere Inzidenzen auf und ab Dezember 2020 stiegen die Inzidenzen einer COVID-19-Erkrankung in Bevölkerungsgruppen mit niedrigem Einkommen deutlich an.

Die folgenden 5 Beiträge widmen sich spezifischen Bevölkerungsgruppen, wobei der erste Beitrag von *Gleich et al.* Sterbefälle in München untersucht. Bei einem Vergleich aller Münchner Todesbescheinigungen im Zeitraum von März bis Dezember 2020 verstarben insgesamt 8,3 % aller Sterbefälle an einer gesicherten COVID-19- und 0,1 % an einer Influenzaerkrankung. Bei einer Untersuchung der Todesbescheinigungen von März bis Juli 2020 zeigte sich, dass ein Viertel der Münchner COVID-19-Sterbefälle im Rahmen nosokomialer Ausbrüche bei hochbetagten, chronisch kranken Bewohner:innen von Pflegeeinrichtungen auftrat (weiterer Beitrag von *Gleich et al.*). Dabei ergaben sich Hinweise auf eine nicht adäquate Risikoeinschätzung und ein unzureichendes Hygienemanagement in den jeweiligen Einrichtungen.

Im Beitrag von *Manz und Mansmann* wurden ebenfalls Sterbekennziffern berechnet, aber die Untersuchung auf ganz Bayern ausgedehnt. Dabei wurde das Konzept der standardisierten Fallfatalitätsrate (sFFR) genutzt, bei der das Verhältnis der regionalen Abweichung in der Mortalität zur regionalen Abweichung im dokumentierten Infektionsprozess beschrieben wird. In den betrachteten Quartalen von April 2020 bis März 2021 ergaben sich heterogene regionale SARS-CoV-2-spezifische sFFR-Werte, die sich zudem über die Zeit veränderten.

Der Beitrag von *Berger et al.* widmet sich speziell dem Aspekt der Einsamkeit als Folge der SARS-CoV-2-Pandemie und der Maßnahmen zu ihrer Eindämmung. Dazu wurden die Teilnehmenden der NAKO-Gesundheitsstudie im Frühjahr 2020 befragt. 31,7 % der NAKO-Teilnehmenden nahmen sich als einsam wahr, wobei Frauen und junge Menschen stärker betroffen waren als Männer und ältere Personen. Zudem nahm mit steigender Einsamkeit der Schweregrad von Depressions- und Angstsymptomen zu.

*Von Felden et al.* wenden sich der durch COVID-19 besonders gefährdeten Gruppe von Mitarbeitenden in Gesundheitseinrichtungen zu. Sie betrachten speziell Mitarbeitende am Universitätsklinikum Hamburg-Eppendorf, die während der 1. Welle PCR-positiv auf SARS-CoV‑2 getestet wurden. Diese berichteten zum größten Teil über milde Verläufe, wobei jedoch einige noch nach Monaten unter Symptomen litten.

Wir wünschen Ihnen eine interessante Lektüre,

Ihre Iris Pigeot, Ihre Sabine Gleich und Ihr Linus Grabenhenrich

## Footnotes

1 Die Arbeit der Herausgeber:innen dieses Hefts wurde durch die NFDI4Health Task Force COVID-19 mit Förderung durch die DFG unterstützt (Projektnummer 451265285, PI 345/17‑1, GR 4686/3-1).

